# The juvenile hormone described in *Rhodnius prolixus* by Wigglesworth is juvenile hormone III skipped bisepoxide

**DOI:** 10.1038/s41598-020-59495-1

**Published:** 2020-02-20

**Authors:** Maria Jose Villalobos-Sambucaro, Marcela Nouzova, Cesar E. Ramirez, María Eugenia Alzugaray, Francisco Fernandez-Lima, Jorge Rafael Ronderos, Fernando G. Noriega

**Affiliations:** 1 Cátedra Histología y Embriología Animal (FCNyM-UNLP), La Plata, Argentina; 2 Department of Biological Sciences and Biomolecular Science Institute, Florida International University, Miami, FL USA; 3 Institute of Parasitology, Biology Centre CAS, Ceske Budejovice, Czech Republic; 4 Department of Chemistry and Biochemistry and Biomolecular Science Institute, Florida International University, Miami, USA; 5 Consejo Nacional de Investigaciones Científicas y Técnicas (CONICET-ARGENTINA), Buenos Aires, Argentina

**Keywords:** Physiology, Zoology

## Abstract

Juvenile hormones (JHs) are sesquiterpenoids synthesized by the *corpora allata* (CA). They play critical roles during insect development and reproduction. The first JH was described in 1934 as a “metamorphosis inhibitory hormone” in *Rhodnius prolixus* by Sir Vincent B. Wigglesworth. Remarkably, in spite of the importance of *R. prolixus* as vectors of Chagas disease and model organisms in insect physiology, the original JH that Wigglesworth described for the kissing-bug *R. prolixus* remained unidentified. We employed liquid chromatography mass spectrometry to search for the JH homologs present in the hemolymph of fourth instar nymphs of *R. prolixus*. Wigglesworth’s original JH is the JH III skipped bisepoxide (JHSB3), a homolog identified in other heteropteran species. Changes in the titer of JHSB3 were studied during the 10-day long molting cycle of 4^th^ instar nymph, between a blood meal and the ecdysis to 5^th^ instar. In addition we measured the changes of mRNA levels in the CA for the 13 enzymes of the JH biosynthetic pathway during the molting cycle of 4^th^ instar. Almost 90 years after the first descriptions of the role of JH in insects, this study finally reveals that the specific JH homolog responsible for Wigglesworth’s original observations is JHSB3.

## Introduction

Sir Vincent B. Wigglesworth, the famous British insect endocrinologist, published a series of three classical manuscripts in the years 1934, 1936 and 1940 that described the existence of an “inhibitory hormone” that prevented metamorphosis in the early nymphal stages of the blood-sucking bug *Rhodnius prolixus*^1–3^. The manuscripts contained a series of elegant studies, including a parabiosis techniques that allowed the mixing of the hemolymph of fourth and fifth instar nymphs, as well as *corpora allata* (CA) transplantation experiments. These studies permitted Wigglesworth to demonstrate for the first time the existence of an “inhibitory hormone” secreted by the CA and delivered to target tissues by the hemolymph. In his latest article, Wigglesworth proposed for the first time to name the “inhibitory hormone” as “juvenile hormone” (JH)^3^. Wigglesworth also used transplantation and parabiosis experiments to reveal that the CA of the 4^th^ instar nymphs of *R. prolixus* was an equally good source of a “yolk-forming hormone”, whereas the CA of a 5^th^ instar nymph was ineffective. He concluded that only a single hormone, the “Juvenile Hormone”, was responsible for both actions, the inhibition of metamorphosis, as well as the control of development of the reproductive organs in the adult^4^.

After the pioneering studies by Wigglesworth and others, the chemical nature of JH remained unidentified until the late 1960’s. The first two JHs, named JH I and II, were isolated from the moth *Hyalophora cecropia*. They were identified as sesquiterpenes, with a methyl ester (α, β-unsaturated) at the C1 position and an epoxide ring at the C10-C11 position^5,6^. Juvenile hormone III, the homolog found in most insects, was described in 1973 from the moth *Manduca sexta*^7^. Finally, two double-epoxidated compounds were later reported, JH III bisepoxide (JHB3) in *Drosophila melanogaster*^8^, and JH III skipped bisepoxide (JHSB3) in the heteropteran *Plautia stali*^9,10^. Surprisingly, in spite of the importance of *R. prolixus*, as a vector of Chagas disease and as a model organism in insect physiology, the identity of Wigglesworth’s original JH remained unknown.

We have previously described the potential of mass spectrometry (MS) based protocols for structural identification and quantification of JH homologues from insect samples^11,12^. More recently, we reported on a liquid chromatography–mass spectrometry (LC-MS/MS) protocol for the identification and quantification of the five most common JH homologs^13^. This protocol allows the detection of JH I, JH II, JH III, JH III bisepoxide (JHB3) and JH III skipped bisepoxide (JHSB3) in a single LC-MS/MS run. In the present study, this modified LC-MS/MS method was utilized to screen the hemolymph of 4^th^ instar nymphs of *R. prolixus*. Our studies revealed that the Wigglesworth’s original JH is JHSB3, a JH homolog previously identified in several heteropteran species^9,13,14^. The changes in the titer of JHSB3 were measured in the hemolymph of *R. prolixus* 4^th^ instar nymphs during the molting cycle, and quantitative real-time PCR (qRT-PCR) studies revealed the changes of mRNA levels in the CA for the 13 enzymes of the JH biosynthetic pathway during the molting cycle of 4th instar nymphs.

## Results

### Identification of jhsb3 from the hemolymph of *R. prolixus*

**Figure 1:**
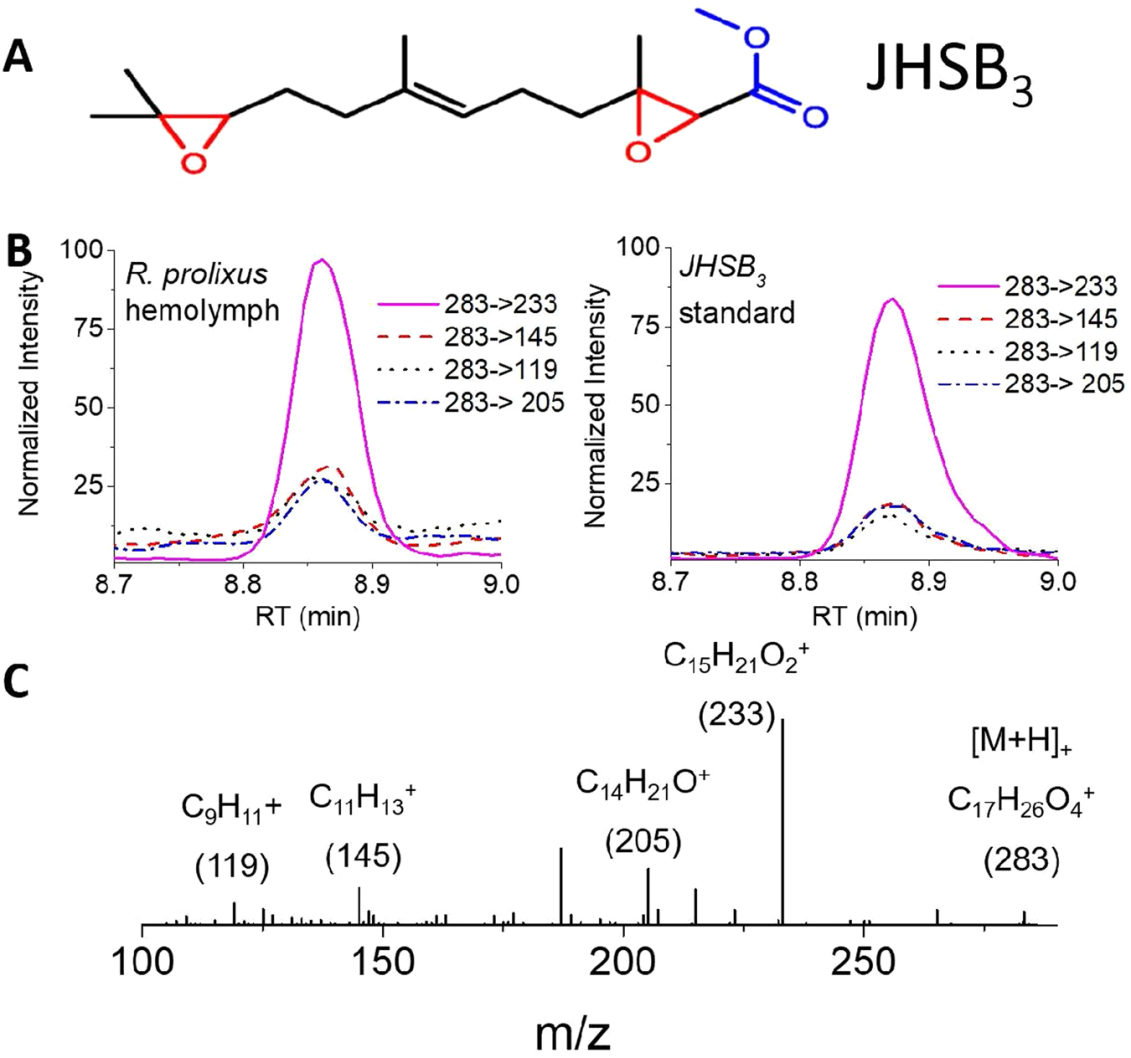
Identification of JHSB3 from the hemolymph of *R. prolixus*. (**A**) Structure of JHSB3. Epoxide groups are in red. The methyl esters group is in blue. (**B**) Typical ion extracted chromatograms of the four transitions (e.g., 283 → 233, 283 → 145, 283 → 119, and 283 → 205) utilized to verify the presence of JHSB3 in the hemolymph (left) and a JHSB3 standard (right). (**C**) Typical ultra-high, resolution FT-ICR MS/MS spectrum of a JHSB3 standard. The parent and the four fragment ions utilized for the experimental JHSB3 verification are labeled in the spectra.

The screening for five JH homologs (e.g., JH I, JH II, JH III, JHB3 and JHSB3) using LC-MS/MS in the hemolymph of 4^th^ instar nymphs of *R. prolixus* revealed only the presence of JHSB3 (Fig. 1A). The method is based on reverse phase LC with electrospray ionization as interface with a triple-quadrupole mass spectrometer. The latter was operated under multiple reaction monitoring (MRM) mode, detecting fragmentation ions after collision-induced dissociation (CID)^13^. While the JH analogs are separated in the LC domain, their unique fragmentation patterns allows for a highly selective detection which provides unequivocal identification and trace-level quantification (Supplemental Table S1). For JHSB3, we screened for four fragmentation channels (e.g., 283 → 233, 283 → 145, 283 → 119, and 283 → 205) in order to increase identification confidence (see comparison between the signal obtained from the *R. prolixus* hemolymph and the JHSB3 standard in Fig. 1B). Each of the fragmentation channels were confirmed by obtaining highly accurate mass measurements from a certified JHSB3 standard analyzed with an ultra-high resolution mass spectrometer (Fig. 1C) (<1 ppm mass error, details in Supplemental Table S2). The m/z signals from 283 corresponds to the protonated pseudomolecular ion ([M + H]^+^; C_17_H_26_O_4_^+^, JHSB3 parent ion) and m/z 233, 205, 145 and 119 correspond to fragment ions C_15_H_21_O_2_^+^, C_14_H_21_O^+^, C_11_H_13_^+^ and C_9_H_11_^+^, respectively. The quantification of the JHSB3 from the *R. prolixus* hemolymph was done using deuterated JH III (JH III-D3) as internal standard, as previously described^13^.

### JHSB3 hemolymph titer during the molting cycle of 4^th^ instar nymphs

**Figure 2:**
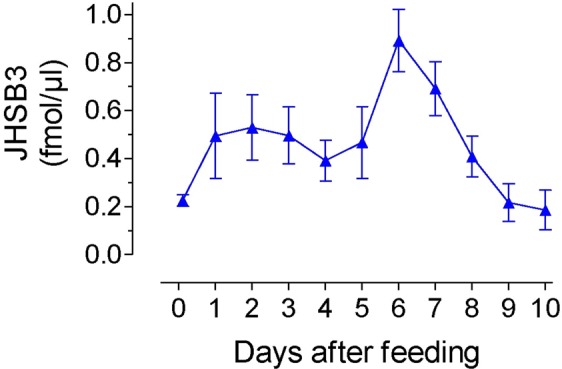
JHSB3 titer in the hemolymph of 4^th^ instar of *R. prolixus*. Nymphs were blood-fed and hemolymph was collected at different days after blood feeding until molting to 5th instar (10 days later). Day 0 represents insects just before feeding. Each data point represents the mean ± SEM of at least four independent replicates of hemolymph of groups of 15 nymphs each.

Hemolymph was obtained from blood-fed 4^th^ instar nymphs every day during the 10 days of the molting cycle, as well as just before blood-feeding. JHSB3 titer was measured using a LC-MS/MS protocol^13^. Addition of deuterated JH III as an internal standard enables the absolute quantification of JHs^13^. The changes in the titer of JHSB3 detected in hemolymph of 4^th^ instar nymph after blood feeding are presented in Fig. 2. Titer was low before feeding and significantly increased by day 2 after blood feeding. It remained relatively constant with a titer around 0.5 fmol/µl until day 5, reaching a peak of around 1 fmol/µl at day 6–7. JHSB3 titer decreased by day 8, and were low until ecdysis.

### Developmental changes in mRNA levels for the JH biosynthetic enzymes

**Figure 3:**
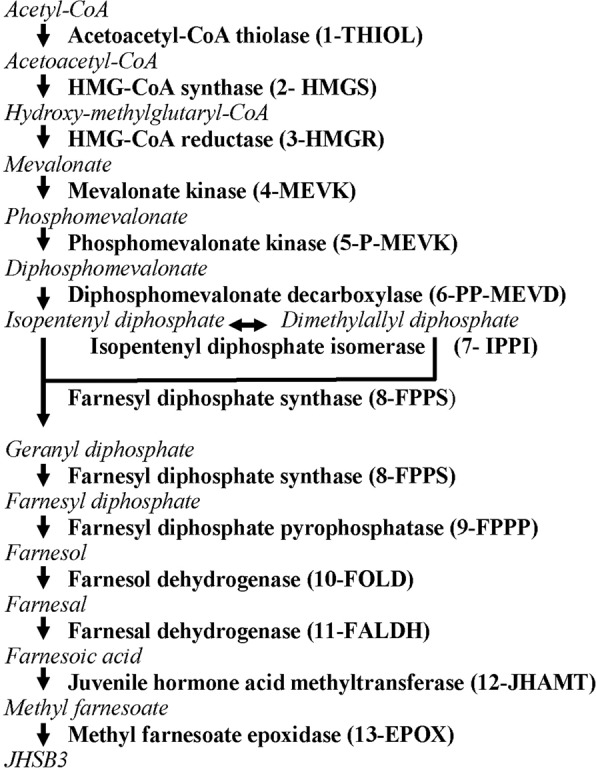
Scheme of JHSB3 biosynthesis. Precursors are in italic and connected by arrows. Enzymes are in bold. Numbers before the enzyme refers to the position in the pathway. Abbreviations for the enzymes are between brackets.

The biosynthetic pathway of JH in the CA of insects includes 13 enzymatic reactions, and it is generally divided into early and late steps (Fig. 3). The early steps follow the mevalonate pathway (MVAP) to form farnesyl pyrophosphate (FPP). In the late steps, often named the JH-specific branch, conversion of FPP to JHSB3 is catalyzed by five enzymes. Annotation of twelve of the thirteen genes encoding the JH-biosynthetic enzymes was described in the original report of the *R. prolixus* genome^15^. To design primers to study the expression of the thirteen enzymes, we reviewed those annotations (Supplemental Table S3). Each of the eight MVAP enzymes seem to be encoded by single-copy genes. Seven of the eight MVAP enzyme genes were correctly predicted. Hydroxymethyl-glutaryl-CoA reductase (HMGR) was not originally annotated in the genome manuscript^15^. Our search revealed the presence of a non-annotated sequences with homology to insect’s HMGRs.

Four of the “late-steps” JH biosynthetic enzymes seem to also be encoded by single-copy genes. Three of these genes were correctly annotated. The original annotation of juvenile acid methyl transferase (JHAMT) was incorrect (RPRC004476). This gene is not expressed in the CA and can therefore be ruled out as being part of the JH biosynthetic pathway. The true JHAMT, identified in this study, is RPRC011659. The search for farnesol dehydrogenase (FOLD) was more complex. The original insect gene, encoding a NADP^+^-dependent short chain dehydrogenase (SDR) was described in *Aedes aegypti*^16^. We found ten SDRs with homology higher than 35% (Supplemental Table S4). Tissue specificity analysis revealed that 4 of them were expressed in the CA (Supplemental Fig. S1). We studied the expression of these 4 genes in the CA during the molting cycle of 4^th^ instar nymph (Supplemental Fig. S2), based on tissue specificity and pattern of expression we selected FOLD 9 for further analysis.

**Figure 4:**
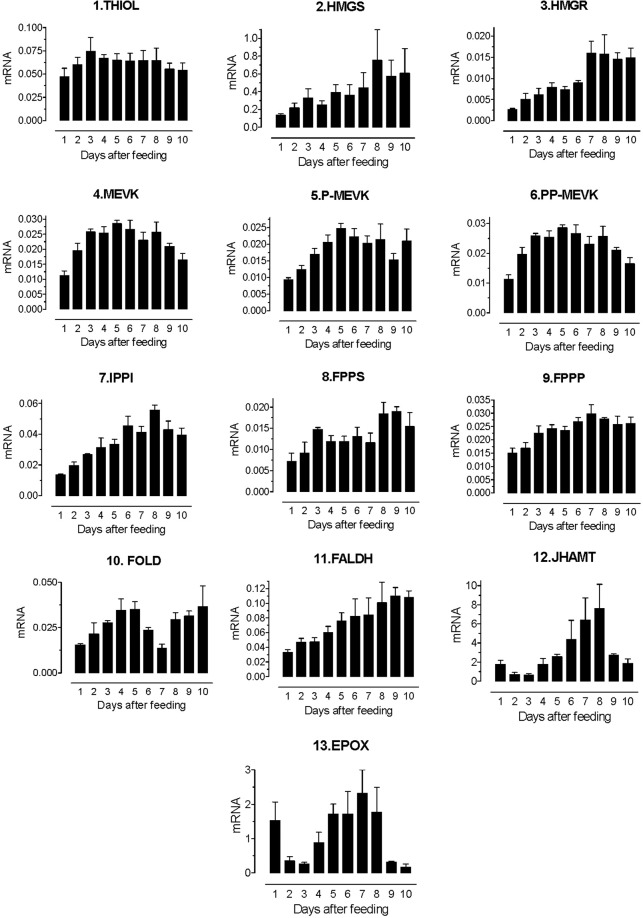
Developmental changes in the expression of JH biosynthetic enzyme mRNAs in the CA. Expression of JH biosynthetic enzymes mRNAs in CA of 4^th^ instar of *R. prolixus*. Nymphs were blood-fed and CAs were dissected at different days after blood feeding until molting to 5^th^ instar (10 days later). Enzyme mRNA bars represent the number of transcripts detected by RT-qPCR, and normalized using the expression of the rpL32 gene. Each RT-PCR data point is average of three independent biological replicates of 15 CA complexes. Enzyme abbreviations are as in Fig. 3.

After the re-annotation of the JH-biosynthetic enzymes, we selected the 13 putative genes to study the expression changes during the molting cycle. The accession numbers and primer sequences for the housekeeping gene 60S ribosomal protein L32 and the 13 enzyme genes are included in Supplemental Table S3. Quantitative real-time PCR (qRT-PCR) confirmed that the 13 genes were expressed in the CA (Supplemental Fig. S3). We compared the levels of expression of the genes encoding the 13 JH biosynthetic enzymes at a time of high expression for most genes (day 8 after blood feeding). There was a 350-fold difference in the levels of mRNA expression in the CA among the genes analyzed (Supplemental Fig. S4). Transcripts for the two last enzymes, JHAMT and epoxidase (EPOX) were the most abundant. The analysis of the expression changes during the molting cycle revealed that transcript levels for most of the enzymes were low during the first 3 days. The maximum relative mRNA levels for most enzymes were detected by day 5–8 after blood feeding (Fig. 4).

## Discussion

### JHSB3 is the circulating hormone in hemolymph of *R. prolixus*

Our studies revealed that the JH originally described in *R. prolixus* by Wigglesworth is JHSB3, a hormone previously identified in four additional species of Hemiptera-Heteroptera. JHSB3 was originally described in *P. stali* (Pentatomomorpha, Pentatomidae)^9^. Later it was reported in *Pyrrhocoris apterus* (Pentatomomorpha, Pyrrhocoridae)^14^. We recently described the presence of JHSB3 in *Oncopeltus fasciatus* (Pentatomomorpha, Pyrrhocoridae) and in the kissing-bug *Dipetalogaster maxima* (Cimicomorpha, Reduvidae)^13^. In the present study the unambiguous identification was based on multiple reaction monitoring analysis using four channels and parallel ultra-high resolution FT-ICR MS/MS confirmation of the purity of the JHSB3 standard and identification of the fragment ions (Fig. 1B,C). It is important to emphasize that the LC-MS/MS protocol not only provided accurate information on the identity of the JH homologs present in the hemolymph of *R. prolixus*, but also ruled out the presence of the additional four JHs surveyed by our method. In a recent study, we analyzed the five JHs present in biological samples from nine different insect species belonging to four orders. JHSB3 was the only JH homolog present in Heteroptera, and it was absent in samples from Diptera, Hymenoptera and Lepidoptera^13^. Pentatomomorpha and Cimicomorpha are two closely related infra-orders in the sub-order Heteroptera^17^. The five Heteropteran species examined up to now belong to these two infra-orders, therefore more studies are needed to conclude if JHSB3 is a homolog exclusively present in all members of the sub-order Heteroptera, as well as in other groups of Hemiptera.

### The JH biosynthetic pathway in *R. prolixus*

We also investigated the JH biosynthetic pathway in *R. prolixus* and compared it to the pathway in other insect taxa. Although the five major JH homologs described in insects have structural differences, they seem to be synthesized through a similar biosynthetic pathway, which includes 13 enzymatic steps^18^. Changes in substrate specificity are responsible for some of the structural differences among JHs. The presence of ethyl substitutions in the Lepidopteran’s JH I and JH II are the result of modifications in the mevalonate pathway, where the thiolase can incorporate the six-carbon building blocks homo-isopentenyl diphosphate and homo-dimethylallyl diphosphate to generate 17 and 18 carbons JHs^19,20^. The enzymes of the MVAP are well conserved in eukaryotes. As previously described in other insects^21^, in *R. prolixus* each of the MVAP enzymes are encoded by a single gene. There is less conservation in the late steps of the pathway^13^. The three enzymes involved in the conversion of FPP to FA are often encoded by families of homolog genes with broad substrate specificity and expression in a wide number of tissues^16,22,23^. In *R. prolixus* we also found several FOLD forms, the homolog depicted in Fig. 4 was expressed in the CA, but confirmation of its role in JH synthesis will require additional studies.

CYP15A1^24^, the cytochrome P450 that catalyzes epoxidation of methyl farnesoate (MF) into JH is also well conserved in insects^25^. Lepidoptera have a CYP15A1 that preferentially converts farnesoic acid (FA) into juvenile hormone acid (JHA), rather than transforming MF into JH^26^, therefore epoxidation precedes methylation. In most other insects there is no epoxidation of FA, but instead an esterification of FA to form MF, followed by epoxidation to JH. Diptera of the suborder Brachycera, such as *Drosophila melanogaster*, synthesize the double-epoxidated JHB3^8^. This “second” epoxidation seems to be a derived trait, and fruit-flies lack a clear ortholog of CYP15A1^24^. Helvig *et al*.^24^ proposed that the CYP15 of higher flies evolved to allow the epoxidation at both the 6, 7 and 10, 11 double bonds, and this evolution resulted in such significant changes that the sequence is no longer recognizable as a CYP15. On the other hand, *R. prolixus* have a typical CYP15A1 epoxidase with very high CA-specific expression. To make JHSB3, kissing-bugs should epoxidate the 2, 3 and 10, 11 double bonds. It would be difficult for a single enzyme to catalyze the formation of the first epoxide group correctly on one of the double bonds of MF, and then move the partially epoxidated product in order to catalyze the second epoxidation; nevertheless to elucidate if Heteroptera have two different epoxidases to generate the 2 distinct epoxide rings will require further investigation.

The analysis of the levels and patterns of expression of the enzymes revealed similarities with previous studies in mosquitoes^27^, silkworm^28,29^, cockroaches^30^ and locusts^31^. In all these species, there were highly significant differences in the expression of the genes encoding the different enzymes, with JHAMT and epoxidase as the most highly expressed genes. In addition, in all these studies, there were significant changes in levels of expression during the life cycles, with increases in the mRNA titer for most enzymes often correlating well with increases in JH synthesis or titer. These two general trends were also observed in our studies in *R. prolixus*, with high mRNA levels of JHAMT and epoxidase overlapping with the major peak of JH titer.

### The major peaks of JH and 20E titer overlap during the molting cycle of 4^th^ instar *R. prolixus*

The molting cycle in *R. prolixus* is initiated by a blood meal, which through the actions of different brain factors, stimulates the synthesis and release of JH and ecdysone^32–35^. The only previous measurements of JH hemolymph titer in *R. prolixus* were performed using a radioimmunoassay (RIA)^33^. It described the presence of two hormonal peaks in 4^th^ instar nymphs; a smaller JH peak on day 1 after a blood meal and a second larger JH peak on day 7. While we only detected JHSB3, this previous study described the presence of JH I, JH II and JH III (the existence of JHSB3 was not known at that time). The identification of these three JH homologs was only based on immuno-reactivities against distinct antibodies. However, the specificity of the different JH-generated RIA antibodies show considerable cross-reactivity among different JHs^36^. It is therefore likely that the JH’s detected in the 1978 Baehr study were actually JHSB3^33^. The second larger peak on day 7 overlapped with the major rise in ecdysteroid titer^33–35^, which induces the molt to 5^th^ instar. Similar to previous work, we also detected a 2-peak JH profile, with a significant increase in JH titer by day 2 after blood feeding, followed by a major peak of JH around day 6–7.

**Figure 5:**
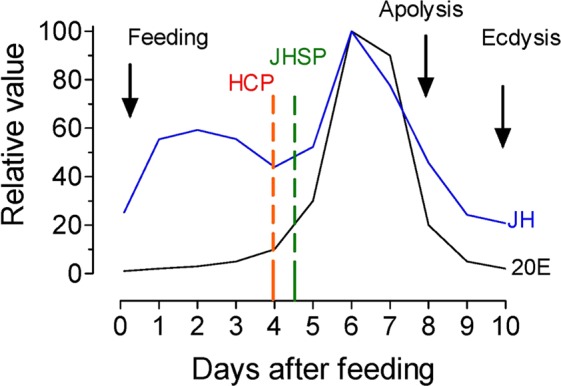
Changes in JH and 20E titer during the molting cycle of 4^th^ instar *R. prolixus*. The changes in JH titer are presented together with 20E titer (from^28–30^), the head critical period (HCP, from^34^), the JH-sensitive period (JHSP from^1^) and the apolysis (from^37^).

Wigglesworth demonstrated that removal of the head of 4^th^ instar nymphs up to 4 days after a blood meal prevented molting. This period of ecdysone synthesis and secretion before becoming independent of the brain is called the ‘head critical period’ (HCP), and it is related to the synthesis of prothoracicotropic hormone (PTTH) by the brain^37^, which stimulates ecdysone synthesis^38^. There is also a “juvenile hormone-sensitive period” for metamorphosis. Removal of the head during this time causes premature metamorphosis^1^. This precocious metamorphosis is caused by interruption of JH signaling^39^. RNAi-mediated knockdown of the JH receptor Methoprene-tolerant (*Met*) in 4^th^ instar *R. prolixus* showed phenotypic alterations characteristic of precocious metamorphosis^40^. The timing of the major peak of CA activity around 6–8 day after a blood meal, has been also suggested by studies performing allatectomies^1,2,32^ or chemically obliterating the CA using precocene, an anti-juvenile hormone compound, which acts directly on the CA of sensitive insects to cause anti-JH effects through necrosis of the glands^41^. Treating 4^th^ instar *R. prolixus*^34^ or *Triatoma infestans*^42^ with precocene caused precocious metamorphosis only if applied early before day 4–5, suggesting again that the major peak of JH was present later during the molting cycle. The changes in JH titer that we observed in the present studies correspond well with the timing of some of the critical events previously described during the molting cycle of the 4^th^ instar nymphs of *R. prolixus* (Fig. 5); including the HCP and the “JH-sensitive period”. The second JH peak overlaps with the major peak of 20-hydroxyecdysone and the detachment of the epidermal cell from the cuticle (apolysis)^43^, which precedes the synthesis and deposition of the new nymphal cuticle, and the ecdysis to the 5^th^ nymphal instar.

## Conclusions

The extensive studies of Wigglesworth using *R. prolixus* as a model, which started in the early 1930s and extended for over 40 years, established the existence and major roles of JH in insects. In the present studies, we finally report that the “inhibitory hormone” present in the “kissing-bug” is JHSB3. In addition, we described the changes in hemolymph titer of JHSB3 in 4^th^ instar nymphs, as well as provided information on the expression of the JH-biosynthetic enzymes. These studies are laying the foundation for additional research to further understand the roles of JH on *R. prolixus* development and reproduction.

## Methods

### Insects

*R. prolixus* were reared at 30 ± 2 °C, with 30% relative humidity and a 12:12 h light-dark period. Newly eclosed 4^th^ instar nymphs were starved during 15 days before offering a blood meal.

### Hemolymph collection

Hemolymph (2 µl from unfed and 4 µl from fed insects) was obtained from nymphs by cutting the legs and pressing gently the abdomen. Clean drops of hemolymph were collected with capillary tubes. For each data point, at least four independent samples of 60 µl of hemolymph were collected from pools of 15 insects each for fed nymphs and a pool of 20 insects for the unfed point. Hemolymph was collected in salinized tubes containing 50 µl of anticoagulant solution (PBS with 10 mM EDTA, 26 mM sodium citrate, 26 mM citric acid and 100 mM glucose). Tubes were kept on ice. The JHD3 internal standard was added immediately after hemolymph collection and before hexane extraction.

### JHSB3 identification and quantification

The JHSB3 amounts present in the hemolymph were quantified using a deuterated JH III analog (JH III-D3) as an internal standard to normalize recoveries during the sample preparation, extraction and analysis steps^13^. Certified standard solutions for the JH homologs and JH III-D3 were obtained from Toronto Research Chemicals (Toronto, Canada). After hemolymph extraction, 10 µL of 6.25 ppb of JH III-D3 in acetonitrile were added to each sample, followed by 600 µL of hexane^13^.

Samples were vortexed for 1 minute, and spun for 5 minutes at 4 °C and 2000 g. The organic phase was transferred to a new silanized vial, dried under nitrogen flow, and stored at −20 °C. Dried extracts were re-suspended in 50 µl of acetonitrile, vortexed 1 minute, transferred to a new silanized vial with a fused 250 µL insert^13^. JH homolog screening was performed using a liquid chromatography coupled to tandem mass spectrometry protocol, as previously described by Ramirez *et al*.^13^. Briefly, 35 µL of water were added to the 50 µL acetonitrile extracts. Injections (70 µL) and liquid chromatography (LC) separations were performed by an Advance UHPLC system (Bruker Daltonics Inc, Billerica, MA, USA), equipped with an Xbridge BEH Phenyl Column (4.6 mm × 150 mm, 3.5 µm) protected by a VanGuard cartridge 3.9 mm × 5 mm, 3.5 µm) (Waters, MA, USA). Column temperature was kept at 30 °C. Gradient separation was performed between 0.1% formic acid in water (mobile phase A) and 0.1% formic acid in acetonitrile (mobile phase B) according to the following program: hold 10% B for 0.25 min, increase to 25% B in 3.75 min, increase to 99% B in 4.00 min and hold for another 4.00 min, return to 10% B in 0.50 min and hold for 2.5 min for a total run time of 15 min^13^. Flow rate was also changed according to the following program: hold 0.8 mL/min for 10.50 min, increase to 1.25 mL/min in 0.20 min and hold for another 1.8 min, decrease to 1 mL/min in 0.10 min and decrease to 0.80 mL/min in 2.4 min. Under these conditions, retention times were 8.9 min and 9.4 min for JHSB3 and JH III-D3, respectively^13^.

Detection was performed by a Bruker EvoQ LC-TQ Elite triple quadrupole mass spectrometer (Bruker Daltonics Inc.) equipped with a heated electrospray ionization (HESI) interface. The instrument was operated under positive mode ionization with multiple reaction monitoring (MRM)^13^. Optimization of MRM collision induced dissociation (CID) energies were performed by infusing 5 mg/L solutions of single JH homologs in 0.1% formic acid in acetonitrile. Heated electrospray ionization (HESI) source parameters were optimized with multiple LC-MS/MS runs using final gradient and flow rate conditions^13^. Source parameters were: Spray voltage 4500 V; Cone temperature: 350 °C; cone gas flow: 20 (arbitrary); Heated probe temperature: 350 °C; probe gas flow: 30 (arbitrary); nebulizer flow: 30 (arbitrary)^13^.

The identification of JHSB3 (based on LC-MS/MS of four channels, Fig. 1) was validated using ultra-high resolution mass spectrometry to obtain accurate masses (<1 ppm mass error) of the parent and fragment ions (Supporting Table S2). Ultra-high resolution FT-ICR MS/MS measurements were performed on a 7T Solarix FT-ICR MS instrument (Bruker Daltonics Inc) in positive ion mode; an electrospray ion source was utilized and mass spectra were collected by co-adding 20, 4 Megaword transients and processed using a half-sine apodization followed by fast-Fourier transform^13^.

### Quantitative real-time PCR (qRT-PCR)

For the study of the developmental changes in mRNA levels for the JH biosynthetic enzymes, groups of 15 *corpora allata-corpora cardiaca* complexes (CA-CC) were dissected in triplicate each day during the molting cycle. For the tissue-specificity studies, groups of 3 pair of ovaries, 3 fat bodies and 10 CA-CC were dissected in triplicate from females 8 day after a blood-meal. Total RNA was extracted using the Norgen Biotek total RNA purification kit (Norgen Biotek, Thorold, ON, Canada). Reverse transcription was carried out using the Verso cDNA kit (Thermo Fisher Scientific, Waltham, MA, USA). Real-time PCR was performed with a 7300 Real Time PCR System using Power SYBR Green PCR Master Mix (Thermo Fisher Scientific). PCR reactions were run in triplicate using 1 µl of cDNA per reaction in a 20 µl volume^44^. Transcript levels were normalized with rpL32 transcript levels in the same sample. Each RT-PCR data point is average of 3 independent biological replicates. The primer sequences and accession numbers for the housekeeping gene 60S ribosomal protein L32 and for the different enzyme genes are included in Supplemental Table S3^45^.

### Statistical analysis

Statistical analyses were performed using the GraphPad Prism Software 3.03 (San Diego, CA, USA). The results are expressed as means ± SEM. Significant differences (P ≥ 0.05) were determined with a one tailed students t-test performed in a pair wise manner or by one-way ANOVA followed by Fisher’s LSD test^44^.

## Supplementary information

Supplementary Information.

## Data Availability

All data generated or analyzed during this study are included in this published article (and its Supplementary Information files).
