# Supplementary material for: The juvenile hormone described in Rhodnius prolixus by Wigglesworth is juvenile hormone III skipped bisepoxide
Source: Sci Rep. 2020 Feb 20;10:3091. doi: 10.1038/s41598-020-59495-1 (PMC7033181; doi:10.1038/s41598-020-59495-1)
Supplement: Supplementary file 1 — Supplementary Information. [file 41598_2020_59495_MOESM1_ESM.pdf]

Supplementary information.

Title: The juvenile hormone described in *Rhodnius prolixus* by Wigglesworth is juvenile hormone III skipped bisepoxide.

Maria Jose Villalobos-Sambucaro[1,5], Marcela Nouzova[2,3], Cesar E. Ramirez[4], María Eugenia Alzugaray[1,5], Francisco Fernandez-Lima[4], Jorge Rafael Ronderos[1#] and Fernando G. Noriega[2#].

[1] Cátedra Histología y Embriología Animal (FCNyM-UNLP), La Plata, Argentina.

[2]Department of Biological Sciences and Biomolecular Science Institute, Florida International University, Miami, FL, USA.

[3]Institute of Parasitology, Biology Centre CAS, Ceske Budejovice, Czech Republic

[4]Department of Chemistry and Biochemistry and Biomolecular Science Institute, Florida International University, Miami, USA.

[5] Consejo Nacional de Investigaciones Científicas y Técnicas (CONICET-ARGENTINA)

# Corresponding authors

Correspondence to: noriegaf@fiu.edu

Supplemental Fig S1. Tissue specificity analysis of FOLD 2, 3, 4 and 9.

Groups of three pairs of ovaries, 3 fat bodies and 10 CA-CC were dissected in triplicate from females 8 days after a blood-meal. RNA extraction, reverse transcription and quantitative real-time PCR were performed as described in the Methods section. PCR reactions were run in triplicate. Transcript levels were normalized with rpL32 transcript levels in the same sample. Each RT-PCR data point is average of three independent biological replicates. Accession numbers for the different enzyme genes are included in Supplemental Table S4. CA: *corpora allata* complexes. FB: fat body. OV: ovaries

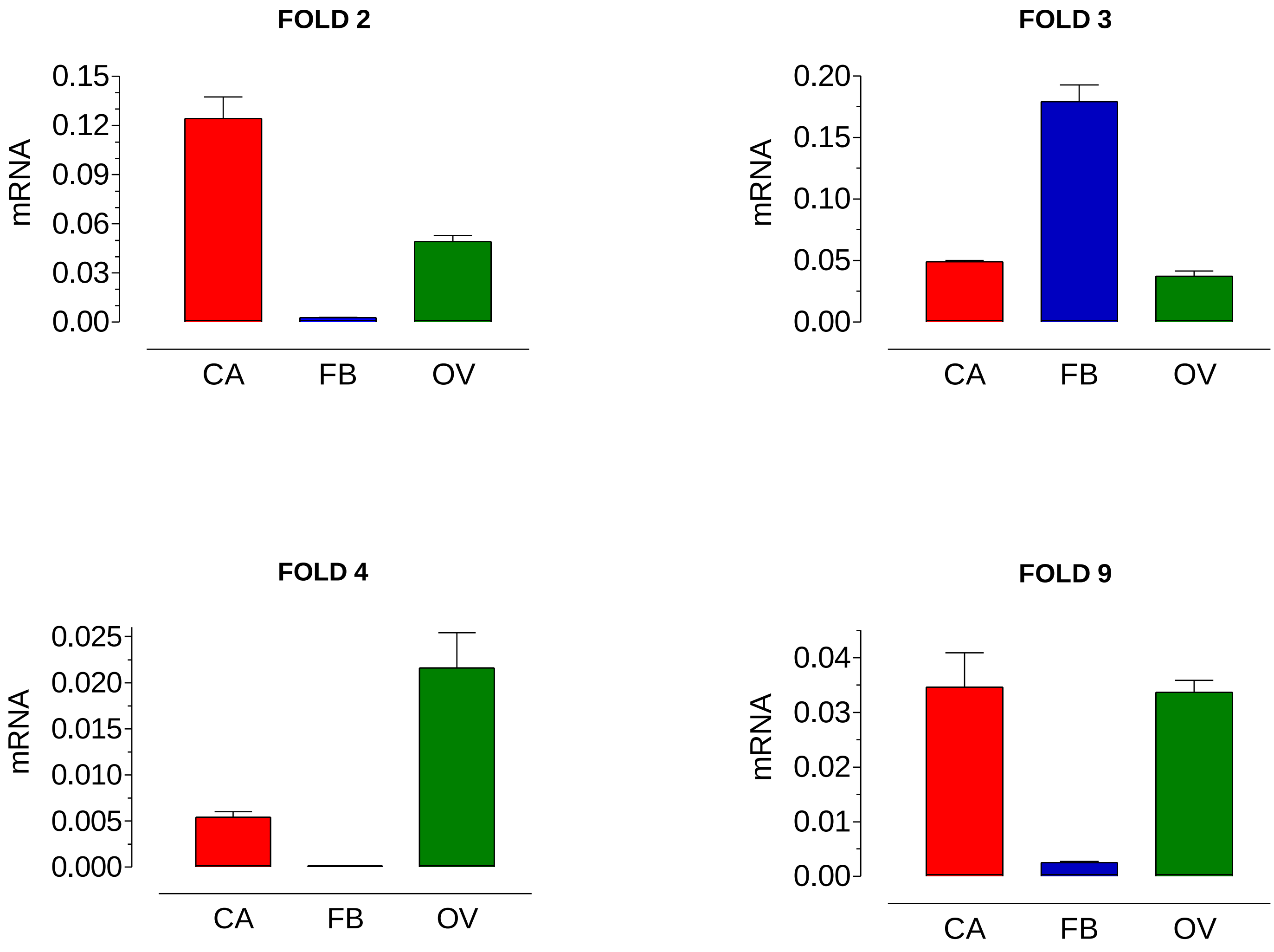

Supplemental Fig. S2. Developmental changes in the expression of FOLD enzymes in the CA. Expression of FOLD enzymes mRNAs in CA of 4th instar of *R. prolixus*. Nymphs were blood-fed and CAs were dissected at different days after blood feeding until molting to 5th instar (10 days later). Enzyme mRNA bars represent the number of transcripts detected by RT-qPCR, and normalized using the expression of the rpL32 gene. Each RT-PCR data point is average of three independent biological replicates of 15 CA complexes.

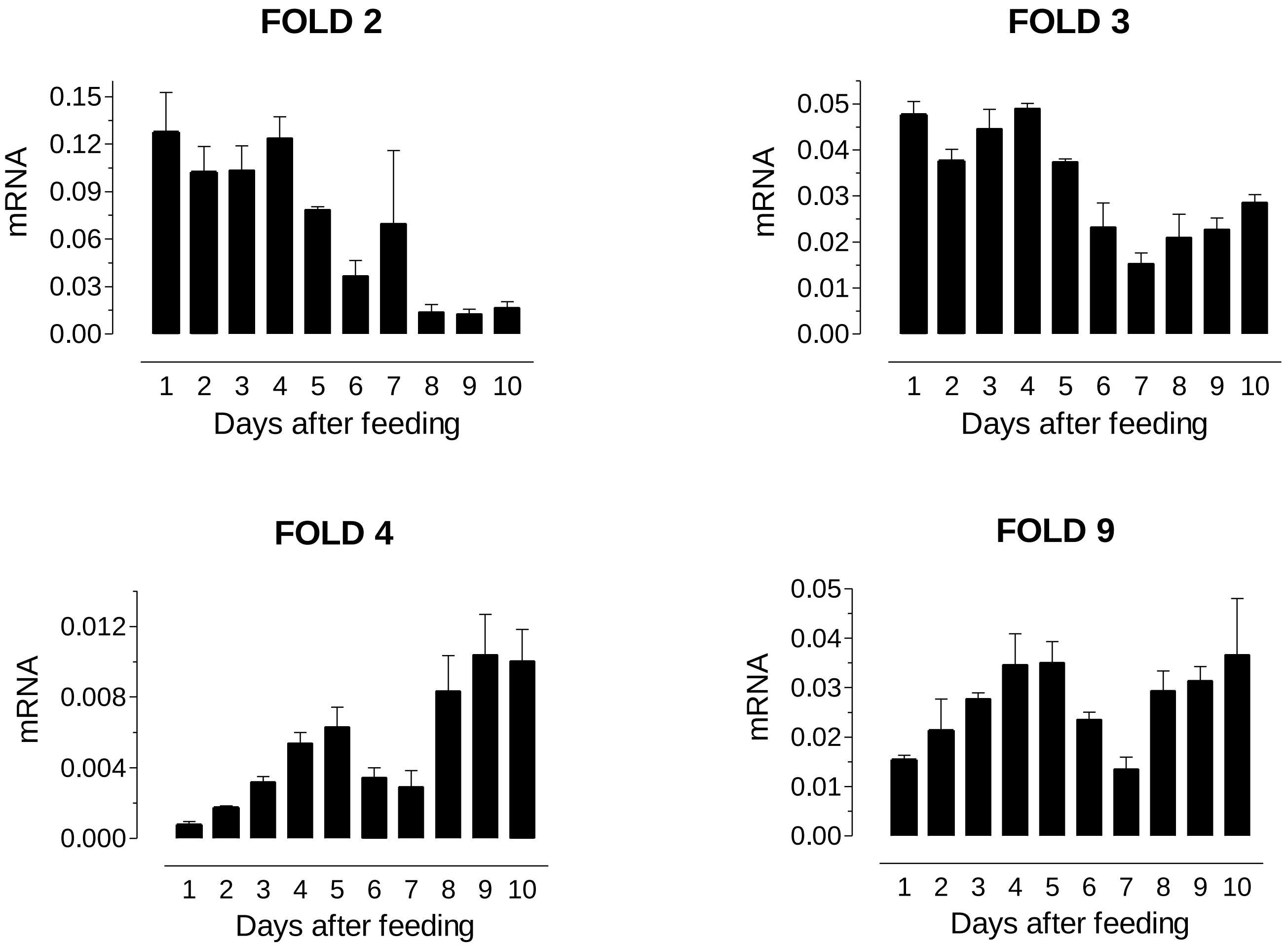

Supplemental Fig S3. **Tissue specificity of the mRNA for the JH biosynthetic enzymes.** Groups of three pairs of ovaries, 3 fat bodies and 10 CA-CC were dissected in triplicate from females 8 days after a blood-meal. RNA extraction, reverse transcription and quantitative real-time PCR were performed as described in the Methods section. PCR reactions were run in triplicate. Transcript levels were normalized with rpL32 transcript levels in the same sample. Each RT-PCR data point is average of three independent biological replicates. The primer sequences and accession numbers for the housekeeping gene 60S ribosomal protein L32 and for the different enzyme genes are included in Supplemental Table S3. CA: *corpora allata* complexes. FB: fat body. OV: ovaries.

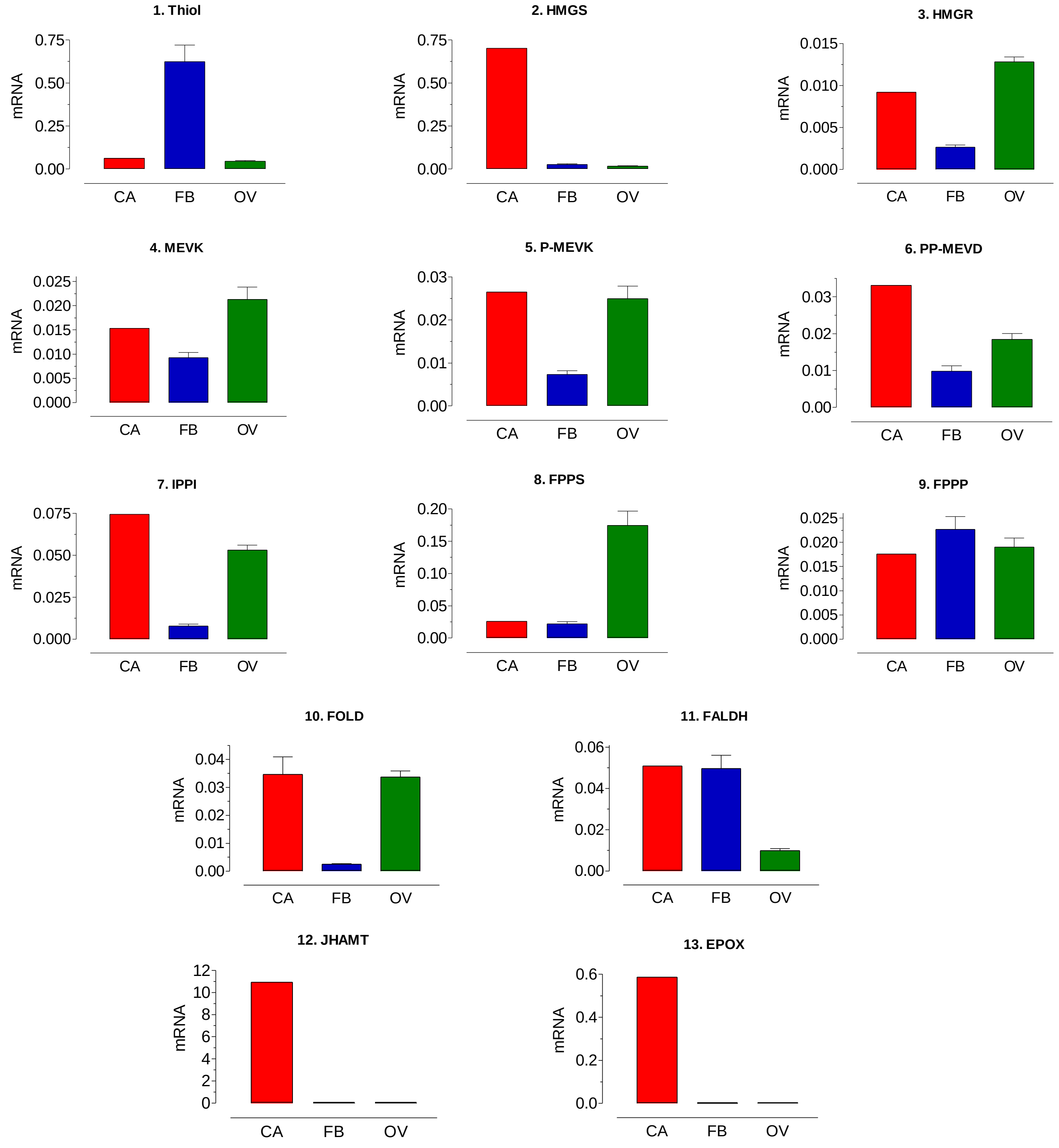

Supplemental Fig. S4. Transcript levels for the JH biosynthetic enzymes in CA of 4th instar *R. prolixus*. Enzymes were grouped in three categories: Lower abundance (blue bars), moderate abundance (red bar) and higher abundance (green bar). Numbers for the enzyme refers to the position in the JH biosynthetic pathway (Fig. 3). Transcript levels were normalized with rpL32 transcript levels in the same sample. Each RT-PCR data point is the average of three independent biological replicates of mRNA from 10 CA-CC dissected from females 8 days after a blood-meal.

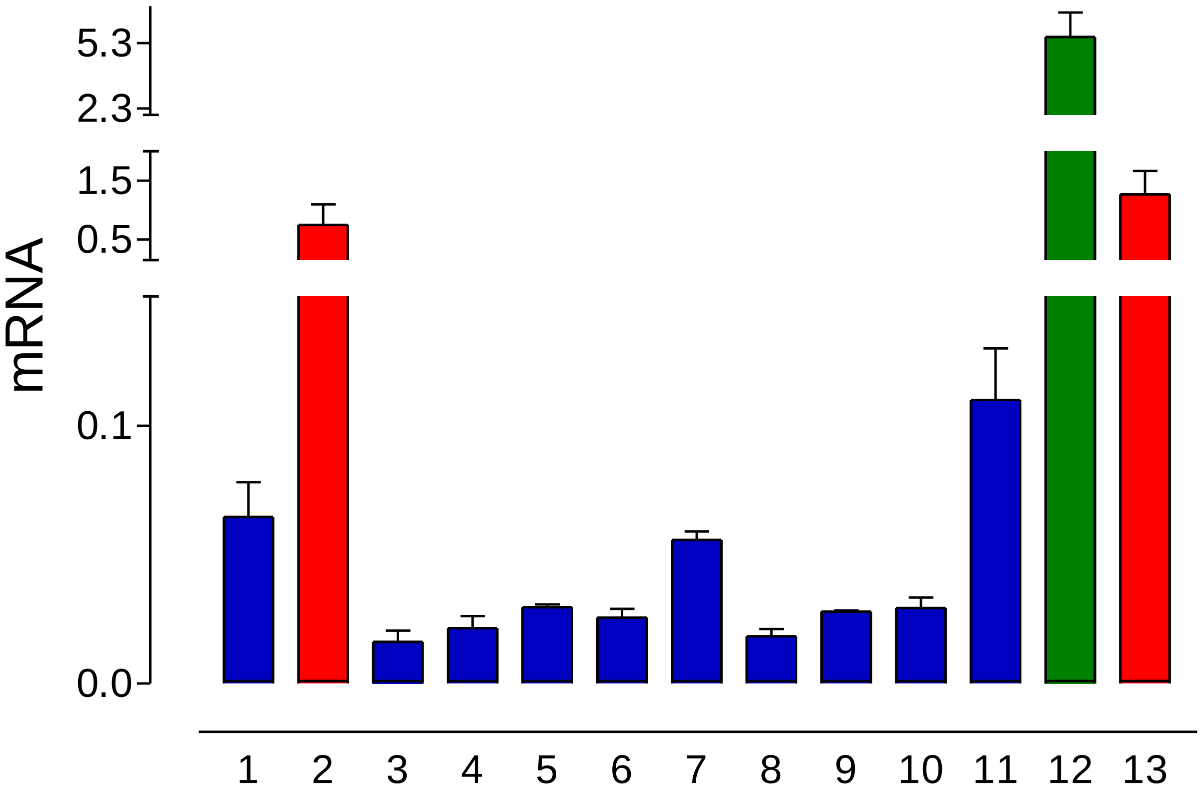

Supplemental Table S1. Parameters for multiple reaction monitoring (MRM) detection of the five most common JH homologs. Qualitative analysis tolerances were ±0.2 min from retention times and ±5% of relative abundances of secondary fragments. LC peak width at base was 0.15 min or lower for all compounds.

	RT (min)	Precursor (m/z)	Main MRM (m/z)	Main MRM CID (V)	2nd MRM (m/z)	2nd MRM CID (V)	3rd MRM (m/z)	3rd MRM CID (V)	4th MRM (m/z)	4th MRM CID (V)
**JHB_3**	8.60	283	233	10.0	251	7.0	-	-	-	-
**$JHSB_3$**	8.94	283	233	9.0	205	12.0	145	18.0	119	18.0
**JH III***	9.40	267	235	5.0	147	10.0	-	-	-	-
**JH II**	9.64	281	249	5.0	231	6.0	-	-	-	-
**JH I**	9.82	295	263	5.0	161	12.0	-	-	-	-

* Detection of JH III-D3 internal standard was done on parent ion m/z 270 with same retention time, fragment ions and collision energies than JH III.

Supplemental Table S2. Ultra-high, resolution FT-ICR MS/MS derived parent and fragment ions of the JHSB3 standard. These four channels were utilized in the identification of JHSB3 in the LC-MS/MS experiments.

m/z	Formula	δ (ppm)
283.19039	$C_{17}H_{26}O_4^+$	0.01
233.15362	$C_{15}H_{21}O_2^+$	0.06
205.15860	$C_{14}H_{21}O^+$	-0.44
145.10110	$C_{11}H_{13}^+$	-0.53
119.08544	C_9H_{11}+	0.73

Supplemental Table S3: Accession # and primer sequences.

Gene	Accession #	Primer forward (5' → 3')	Primer reverse (5' → 3')
**Acetyl-CoA-thiolase**	RPRC007496	FW: CAAAGTTAATGTACACGGTGGTG	RV: CTCCAGACTTCAACGCTGTTA
**HMG-CoA reductase**	Supercontig RproC3:KQ034226 minus strand 878233-875494	FW: GGCATAGAAAGAAGATGACCAAAC	RV: GCACGAGTATCAAGACAACAATATG
**HMG-CoA synthase**	RPRC007884	FW: GCAACTGTTTGAAGAAAGTGGTA	RV: AAGCACTGGTACCTCCAAAG
**Mevalonate Kinase**	RPRC014277	FW: GAAAGATCAAGAGGAACGAGGAG	RV: CGCTTATGTGAGACACCTAATGAT
**Phosphomevalonate Kinase**	RPRC006212	FW: AAATCGTTTCTGACGAACAAGTG	RV: GCAATGACAACATCCCATTCAG
**Diphosphomevalonate decarboxylase**	RPRC012093	FW: CGTGGCCTTCCAGTTCAA	RV: GTATTTGAGGAGACCAGGTTCG
**Isopentenyl diphosphate isomerase**	RPRC003718	FW: CACCAATTACGCCTTGGTTTAG	RV: GTGGATATTCACGTGGTCTTGA
**Farnesyl diphosphate synthase**	RPRC014226	FW: CGCAGTAGTTGCAATGCATAAAG	RV: GCTTCTTGACAGCGGCTATT
**Farnesyl diphosphate pyrophosphatase**	RPRC004412	FW: GCTTGAATCCTAGAAGAGCGTTA	RV: ACCGGTAAGTACAAGCAATGTAT
**Farnesol dehydrogenase**	RPRC010547-RA	FW: AAACCGAGCGATGTTGT	RV: GTAGGTTGGATAACTAGTTCTGAT
**Farnesal dehydrogenase**	RPRC002910	FW: AGTACCTTACAGTCTAGTATTTGCC	RV: GATCTGTCTTCAGCACCGTT
**Juvenil hormone acid methyltransferase**	RPRC011659-RA	FW: GGACCAGGCGATGTTACTTT	RV: CCAAATCATCAGAAATATCGCTTCC
**Methyl farneseoate epoxidase**	RPRC000513	FW: CGGAGAATTGATTCATGATGATTGG	RV: GTAACGGCGGTGACAGTAAA
**L32**	RPRC014419	FW: ACATGCTTCCTACTGGTTTCA	RV: GACACACCATGCGCTATCT

Supplemental Table S4: Information on FOLD genes.

Sequence	Sequence	BLAST Score	Expressed in CA	Contig
Aedes_SDR1	RPRC010112-RA|1-720 RprSDR1	45	-	KQ034192
Aedes_SDR1	RPRC014554-RA|1-729 RprSDR2	41	+	KQ034087
Aedes_SDR1	RPRC011354-RA|1-726 RprSDR10	40	N/A	KQ034121
Aedes_SDR1	RPRC014549-RA|1-750 RprSDR3	42	+	KQ034087
Aedes_SDR1	RPRC014553-RA|1-723 RprSDR4	42	+	KQ034087
Aedes_SDR1	RPRC011377-RA|37-744 RprSDR5	36	-	KQ034121
Aedes_SDR1	RPRC011360-RA|1-708 RprSDR6	38	-	KQ034121
Aedes_SDR1	RPRC007083-RA|1-750 RprSDR7	35	-	KQ034115
Aedes_SDR1	RPRC011398-RA|34-561 RprSDR8	36	-	KQ034121
Aedes_SDR1	RPRC010547-RA|82-510 RprSDR9	43	+	KQ034899

In the Supplemental Table S4are included the 10 sequences with highest scores after BLAST with the FOLD from *Aedes aegypti* (Ae-SDR1), which is a CA farnesol dehydrogenase. Reference #16: Mayoral, J.G, Nouzova, M., Navare, A. & Noriega, F.G. NADP+-dependent farnesol dehydrogenase, a corpora allata enzyme involved in juvenile hormone synthesis *Proc Natl Acad Sci USA* **106**, 21091-21096 (2009).

The *Rhodnius* homologs are named RprSDRs.

Column 2 shows the accession numbers and names of the genes.

Column 3 shows the BLAST score (similarity).

Column 4 shows the expression of the genes in CA measured by qRT-PCR (see manuscript). RprSDR2, 3, 4 and 9 are expressed in CA.

Column 5 shows the contig location in the *R. prolixus* genome. RprSDR2, 3 and 4 are present in the same contig location. They are probably the product of gene duplication events. Based on the expression patterns, RprSDR4 or RprSDR9 are most likely the FOLD expressed in the CA that is part of the JH biosynthesis pathway.

RprSDR5, 6, 8 and 10 are present in the same contig location. RprSDR5, 6 and 8 are not expressed in the CA, so although we did not study the expression of RprSDR10 in CA (N/A: not analyzed), this gene is not expressed in CA.
